# Association of Immunodeficiency and HIV Viremia With Cervical Precancer and Cancer Risk Among Women With HIV in South Africa

**DOI:** 10.1093/cid/ciag093

**Published:** 2026-02-14

**Authors:** Yann Ruffieux, John Andoh, Andreas D Haas, Chido Chinogurei, Katayoun Taghavi, Cari van Schalkwyk, Matthias Egger, Naomi Folb, Gary Maartens, Eliane Rohner

**Affiliations:** Institute of Social and Preventive Medicine, University of Bern, Bern, Switzerland; Institute of Social and Preventive Medicine, University of Bern, Bern, Switzerland; Graduate School for Health Sciences, University of Bern, Bern, Switzerland; Institute of Social and Preventive Medicine, University of Bern, Bern, Switzerland; Centre for Integrated Data and Epidemiological Research, School of Public Health, University of Cape Town, Cape Town, South Africa; Centre for Integrated Data and Epidemiological Research, School of Public Health, University of Cape Town, Cape Town, South Africa; Institute of Social and Preventive Medicine, University of Bern, Bern, Switzerland; Early Detection, Prevention and Infections Branch, International Agency for Research on Cancer, World Health Organization (IARC/WHO), Lyon, France; South African Centre for Epidemiological Modelling and Analysis (SACEMA), Centre for Epidemic Response and Innovation (CERI), School for Data Science and Computational Thinking, Stellenbosch University, Stellenbosch, South Africa; Institute of Social and Preventive Medicine, University of Bern, Bern, Switzerland; Population Health Sciences, Bristol Medical School, University of Bristol, Bristol, United Kingdom; Department of Infectious Diseases and Hospital Epidemiology, University Hospital Zurich, University of Zurich, Zurich, Switzerland; Medscheme, Cape Town, South Africa; Division of Clinical Pharmacology, Department of Medicine, University of Cape Town, Cape Town, South Africa; Division of Clinical Pharmacology, Department of Medicine, University of Cape Town, Cape Town, South Africa; Institute of Social and Preventive Medicine, University of Bern, Bern, Switzerland

**Keywords:** cervical cancer, HIV, CD4 cell count, HIV RNA, South Africa

## Abstract

**Background:**

Women with human immunodeficiency virus-1 (HIV) (WWH) have a higher cervical cancer risk than women without HIV. The timing and extent to which HIV viremia and immunodeficiency contribute to cervical carcinogenesis remain incompletely understood.

**Methods:**

We conducted a cohort study using medical claims data from a South African HIV program (2011–2022) and calculated incidence rates of cervical precancer and cancer. Cox proportional hazards models assessed associations with CD4 cell count and HIV ribonucleic acid (RNA). We tested summary and point-in-time CD4 and HIV RNA measures with 6–36 months lag periods. Models were adjusted for age, calendar year, and antiretroviral therapy (ART) initiation; fully adjusted models included both CD4 count and HIV RNA.

**Results:**

Over 66 000 WWH contributed more than 300 000 person-years; 1202 WWH developed moderate dysplasia (incidence rate: 394/100 000 person-years), 1237 severe dysplasia (405/100 000 person-years), 211 carcinoma in situ (66/100 000 person-years), and 257 cervical cancer (70/100 000 person-years). Lower CD4 counts were strongly associated with higher rates of cervical dysplasia, carcinoma in situ, and cancer, independent of HIV RNA. Lowest CD4 count over 30 months was the most informative measure for cervical dysplasia while CD4 count lagged by 24 months was most informative for cervical cancer. Higher HIV RNA was associated with increased risk of precancer and cancer in models unadjusted for CD4 count, with associations attenuated after adjustment.

**Conclusions:**

Maintaining high CD4 counts and achieving viral suppression through early ART initiation, along with using CD4 cell count for risk stratification in cervical screening, may help improve cervical cancer prevention among WWH in South Africa.

## Teaser

Low CD4 counts were associated with increased rates of cervical precancer and cancer among women with HIV in South Africa. Maintaining high CD4 counts through early initiation of antiretroviral therapy may help prevent cervical cancer in this population.

## Graphical Abstract

**Graphical Abstract:**
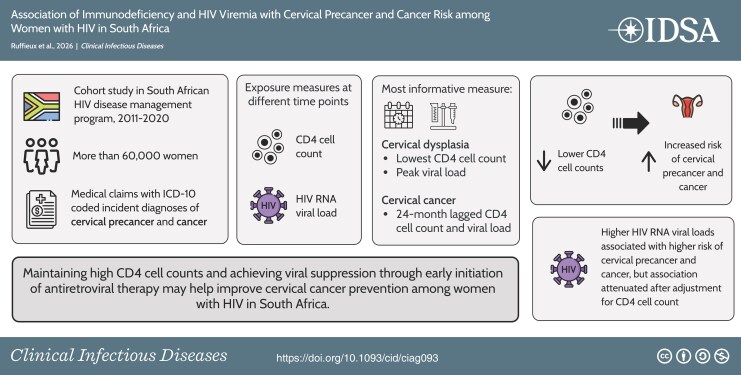
This graphical abstract is also available at Tidbit: https://tidbitapp.io/institutional-portal/clinical-infectious-diseases/tidbits/association-of-immunodeficiency-and-hiv-viremia-with-cervical-precancer-and-cancer-risk-among-women-with-hiv-in-south-africa-bce48f48-4788-4897-9904-39d152cb6f6e/update

Most cervical cancers are caused by oncogenic (high-risk) human papillomavirus (HPV) infection, which can induce histological changes in the cervix [1]. Women with human immunodeficiency virus-1 (HIV) (WWH) are more likely to acquire high-risk HPV, less likely to clear the infection, and experience a faster progression to precancer and invasive disease [2]. Consequently, WWH face a higher risk of developing cervical cancer than women without HIV [2, 3]. Of note, in Southern African countries, most cervical cancer diagnoses occur in WWH [3]. While the elevated risk of cervical cancer in WWH is well established, the role of HIV viremia and immunodeficiency in cervical carcinogenesis remains poorly understood, particularly with respect to the timing and magnitude of their effects. Additionally, while antiretroviral therapy (ART) has dramatically improved survival and quality of life for people with HIV [4], its impact on cervical cancer risk remains unclear [5].

Studies from Europe and North America have consistently shown that lower CD4 counts are associated with a higher cervical cancer risk in WWH. The French Hospital Database on HIV (FHDH) found that the current CD4 count was most strongly associated with cervical cancer [6]. In contrast, the Swiss HIV Cohort Study (SHCS) [7] and the North American AIDS Cohort Collaboration on Research and Design (NA-ACCORD) [8] found that earlier CD4 counts (at baseline, or the nadir count) were most strongly associated with cervical cancer. Data from Southern Africa and other resource-limited settings are scarce. The large South African HIV Cancer Match study [9] modeled cervical cancer incidence as a function of the CD4 count from 1 year before and found a strong association between lower CD4 counts and higher cervical cancer incidence rates, but no clear association with HIV ribonucleic acid (RNA) [10, 11]. Given the high burden of HIV and cervical cancer in countries such as South Africa [12], improving the understanding of these relationships is essential, particularly in the context of the World Health Organization's global initiative to eliminate cervical cancer as a public health problem [13].

In a previous study, we used reimbursement claims from a large South African medical insurance scheme to analyze the incidence of cervical precancer and cancer across the life course [14]. Here, we extend these analyses to examine the association of various lagged, point-in-time, and summary measures of CD4 counts and HIV RNA with moderate and severe cervical dysplasia, cervical carcinoma in situ, and invasive cervical cancer using reimbursement claims data from a private-sector HIV disease management program.

## METHODS

### Data Source

We used data from Aid for AIDS (AfA), South Africa's largest private-sector HIV disease management program, established in 1998 and administered by Medscheme [15]. Aid for AIDS enrolls insured individuals once an HIV diagnosis is confirmed. Antiretroviral therapy is prescribed according to national guidelines, and individuals are followed through linked pharmacy, laboratory, and clinical claims. The claims data were coded based on the International Classification of Diseases (ICD-10), the Anatomical Therapeutic Chemical Classification System, the Current Procedural Terminology, and the National Reference Price List.

### Inclusion Criteria and Definitions

We included women aged ≥18 years who were members of the AfA program at some point between 1 January 2011 and 1 December 2022. The study population varied depending on the outcome of interest and on the measures and time lag used for CD4 count and HIV RNA. We considered 4 outcomes based on ICD-10 codes: moderate cervical dysplasia (N87.1), severe cervical dysplasia (N87.2), cervical carcinoma in situ (D06), and cervical cancer (at least 2 C53 codes). We analyzed each outcome separately and only considered the first occurrence of a given outcome [16]. We excluded women with a single C53 code from the cervical cancer analysis, as claims-based algorithms identifying cervical cancer have been shown to have low specificity when based on single diagnoses [17]. We included women with a single C53 code in a sensitivity analysis. We excluded women diagnosed with the outcome of interest before baseline from the analysis of that outcome, where a woman's baseline date was defined as the latest from their AfA enrollment, 1 January 2011, or her 18th birthday. We also excluded women who did not have at least 2 CD4 count and at least 2 HIV RNA measurements before the end of their person-years at risk.

For each woman, person-years-at-risk started at whichever occurred last: the baseline date, *X* months after the first CD4 count, or *Y* months after the first HIV RNA. The values of *X* and *Y* depended on the lag (varying between 6 and 36 months, as explained below) and the measure used. Person-years-at-risk ended at whichever came first: the first claim with an ICD-10 diagnosis of the outcome of interest, death, exit from the AfA program, or 1 December 2022. If due to the lag applied to CD4 count and HIV RNA measurements women's risk periods would have ended before it could begin, we excluded them from the analysis. Larger values of *X* or *Y* resulted in more exclusions.

### Statistical Analysis

We used descriptive statistics to assess sociodemographic and clinical characteristics of the study population at baseline and at diagnosis of each outcome. We calculated crude incidence rates per 100 000 person-years for each outcome by dividing the number of women with an incident outcome by the total number of person-years-at-risk. We used Cox proportional hazards models to estimate hazard ratios (HRs) for the association of CD4 count and HIV RNA with each of the 4 outcomes. CD4 counts and HIV RNA were lagged by varying intervals, and we considered both summary and point-in-time measures (see Model Selection section). We coded CD4 count and HIV RNA as categorical variables (<50, 50–99, 100–199, 200–349, 350–499, and ≥500 cells/μL; <50, 50–999, 1000–99 999, and ≥100 000 copies/mL). We considered (1) unadjusted models for CD4 count and HIV RNA; (2) separate models for CD4 count and HIV RNA adjusted (based on a priori selection) for age (time-updated; categories 18–34, 35–44, 45–59, and ≥60 years), calendar year (time-updated; categories 2011–2013, 2014–2016, 2017–2019, and 2020–2022), and having initiated ART (yes/no; time-updated); and (3) fully adjusted models combining both CD4 count and HIV RNA in addition to the above factors. CD4 count and HIV RNA were time-updated at monthly intervals, with linear interpolation used to assign values at each time point. Human immunodeficiency virus RNA measurements below the detection limit were set to 20 copies/mL. To assess collinearity in the Cox regressions, we computed generalized variance inflation factors (GVIFs) for CD4 count and HIV RNA. We assessed the proportional hazards assumption for CD4 count and HIV RNA using Schoenfeld residual tests and visual inspection of the log–log plot of the survival functions. There was no clear evidence of this assumption being violated for any of the outcomes.

### Model Selection

For each outcome, we considered lags of 6, 12, 18, 24, 30, and 36 months for point-in-time CD4 count and HIV RNA (the *X* and *Y* introduced above). Additionally, we considered summary measures in a 30-month window, lagged by 6 months (leading to *X* or *Y* = 36 months): lowest CD4 count, average CD4 count, percent time with CD4 counts < 200 cells/μL, peak HIV RNA, average Log10 HIV RNA, and percent time with HIV RNA > 50 copies/mL. We fitted separate adjusted Cox models for each of these measures and chose the model leading to the lowest Akaike information criteria (AIC). The AIC favors the goodness of fit of a model while penalizing further parameter inclusion. For these model comparisons, we restricted the population and person-years-at-risk to a common set per outcome, corresponding to *X* = *Y* = 36 months in the definition of person-years-at-risk. The AICs resulting from fitting the different models are shown in Supplementary Table 1.

## RESULTS

### Study Population and Incidence Rates

**Figure 1.**
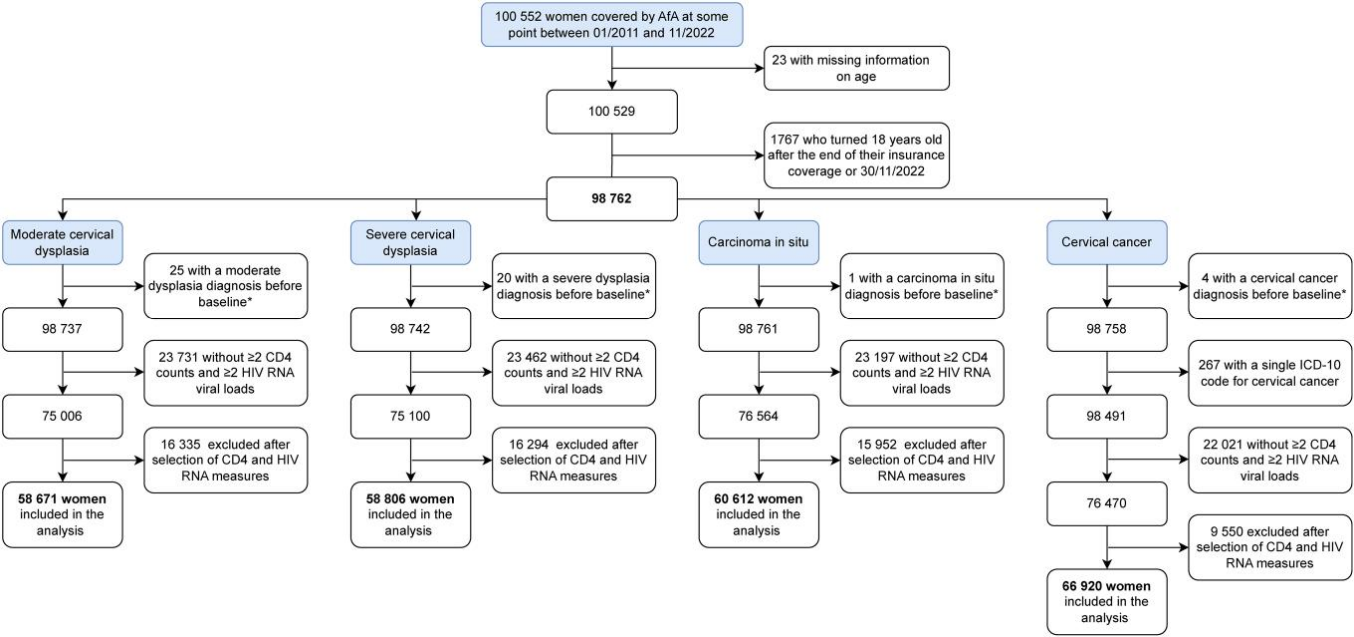
Selection of study population for each outcome. Abbreviations: AfA, Aid for AIDS; HIV, human immunodeficiency virus; ICD-10, International Classification of Diseases; RNA, ribonucleic acid. * Baseline = whichever comes last from 1 January 2011, AfA enrollment, and 18th birthday.

Of 100 552 women covered by AfA at some point between 1 January 2011 and 1 December 2022, we excluded 1767 who had either missing information on age or were aged < 18 years while in the AfA program. Further exclusions depended on the outcome of interest (Figure 1). For example, the study population for the analysis of cervical cancer consisted of 66 920 women, of whom 257 were diagnosed with cervical cancer. The baseline characteristics of women in the study population are shown in Table 1. Women diagnosed with cervical cancer were older at diagnosis than those diagnosed with cervical precancer (Table 2). In Supplementary Figure 1, we show the CD4 count and Log10 HIV RNA trajectories for a random sample of 100 women, along with the median trajectory for the study population.

**Table 1.** Baseline Characteristics of Women Included in the Analysis of Cervical Cancer

Characteristic	Included Women (N = 66 920)
Median age (IQR) [y]	34.8 (29.3, 41.4)
Calendar year, n (%)	
2011–2013	54 016 (80.7)
2014–2016	7801 (11.7)
2017–2019	4302 (6.4)
2020–2022	801 (1.2)
CD4 cell count [cells/µL], n (%)	
0–99	5980 (8.9)
100–199	7338 (11.0)
200–349	14 252 (21.3)
350–499	13 361 (20.0)
≥500	25 989 (38.8)
Median (IQR)	411 (238, 629)
HIV RNA [copies/mL], n (%)	
0–49	25 175 (37.6)
50–9999	6537 (9.8)
10 000–99 999	24 526 (36.6)
≥100 000	10 682 (16.0)
Median (IQR)	1860 (20, 42 426)
Initiated ART before or during years-at-risk, n (%)	64 389 (96.2)

Numbers varied slightly when analyzing the other cervical disease outcomes.

Baseline = latest from 1 January 2011, AfA enrollment, and 18th birthday.

Abbreviations: AfA, Aid for AIDS; ART, antiretroviral therapy; HIV, human immunodeficiency virus; IQR, interquartile range; RNA, ribonucleic acid.

**Table 2.** Characteristics of Women Diagnosed With One of the Four Cervical Disease Outcomes, at the Time of the Diagnosis

	Moderate Dysplasia	Severe Dysplasia	Carcinoma In Situ	Cervical Cancer
Total number	1202	1237	211	257
Median age (IQR) [y]	39.8 (35.6, 44.5)	40.7 (36.1, 46.0)	41.6 (37.1, 48.5)	45.4 (40.4, 50.5)
Calendar year, n (%)				
2011–2013	115 (9.6)	120 (9.7)	32 (15.2)	22 (8.6)
2014–2016	288 (24.0)	303 (24.5)	44 (20.9)	52 (20.2)
2017–2019	494 (41.1)	468 (37.8)	78 (37.0)	100 (38.9)
2020–2022	305 (25.4)	346 (28.0)	57 (27.0)	83 (32.3)
CD4 cell count [cells/µL], n (%)				
0–99	44 (3.7)	35 (2.8)	9 (4.3)	14 (5.4)
100–199	76 (6.3)	72 (5.8)	17 (8.1)	20 (7.8)
200–349	153 (12.7)	144 (11.6)	31 (14.7)	35 (13.6)
350–499	200 (16.6)	229 (18.5)	34 (16.1)	42 (16.3)
≥500	729 (60.6)	757 (61.2)	120 (56.9)	146 (56.8)
Median (IQR)	585 (368, 781)	581 (386, 803)	546 (342, 837)	559 (343, 777)
HIV RNA [copies/mL]^a^, n (%)				
0–49	856 (71.2)	888 (71.8)	153 (72.5)	187 (72.8)
50–9999	131 (10.9)	137 (11.1)	24 (11.4)	23 (8.9)
10 000–99 999	178 (14.8)	174 (14.1)	26 (12.3)	33 (12.8)
≥100 000	37 (3.1)	38 (3.1)	8 (3.8)	14 (5.4)
Median (IQR)	20 (20, 100)	20 (20, 91)	20 (20, 72)	20 (20, 90)
Initiated ART, n (%)	1118 (93.0)	1156 (93.5)	198 (93.8)	237 (92.2)

Abbreviations: ART, antiretroviral therapy; HIV, human immunodeficiency virus; IQR, interquartile range; RNA, ribonucleic acid.

^a^HIV RNA below the detection limit were set to 20 copies/mL.

For each outcome, the person-years-at-risk, incidence rates, and selected CD4 count and HIV RNA measurements are summarized in Table 3. Of note, differences in AIC values across some of the CD4 count and HIV RNA specifications were modest, particularly for carcinoma in situ and cervical cancer (Supplementary Table 1). The incidence rate per 100 000 person-years-at-risk was 394 (95% confidence interval [CI] 372–417) for moderate cervical dysplasia, 405 (95% CI 383–428) for severe dysplasia, 66 (95% CI 58–76) for carcinoma in situ, and 70 (95% CI 61–79) for cervical cancer.

**Table 3.** Measures Chosen by Outcome for CD4 Cell Count and HIV RNA and Incidence Rates per 100 000 Person-Years

Outcome	CD4 Cell Count Measure	HIV RNA Measure	Number of Women With Outcome	Total Number of Women	Person-Years-at-Risk	Median Person-Years [IQR]	Incidence Rate Per 100 000 Person-Years (95% CIs)
Moderate cervical dysplasia	30-m lowest, 6-m lag	30-m peak, 6-m lag	1202	58 671	304 739	4.5 [2.0–8.0]	394 (372–417)
Severe cervical dysplasia	30-m lowest, 6-m lag	30-m peak, 6-m lag	1237	58 806	305 509	4.5 [2.0–8.0]	405 (383–428)
Cervical carcinoma in situ	36-m lag	30-m average, 6-m lag	211	60 612	318 945	4.5 [2.1–8.1]	66 (58–76)
Cervical cancer	24-m lag	24-m lag	257	66 920	369 379	5.0 [2.2–8.5]	70 (61–79)

Abbreviations: CI, confidence interval; HIV, human immunodeficiency virus; IQR, interquartile range; RNA, ribonucleic acid.

### Associations With CD4 Cell Count and Human Immunodeficiency Virus Ribonucleic Acid

**Figure 2.**
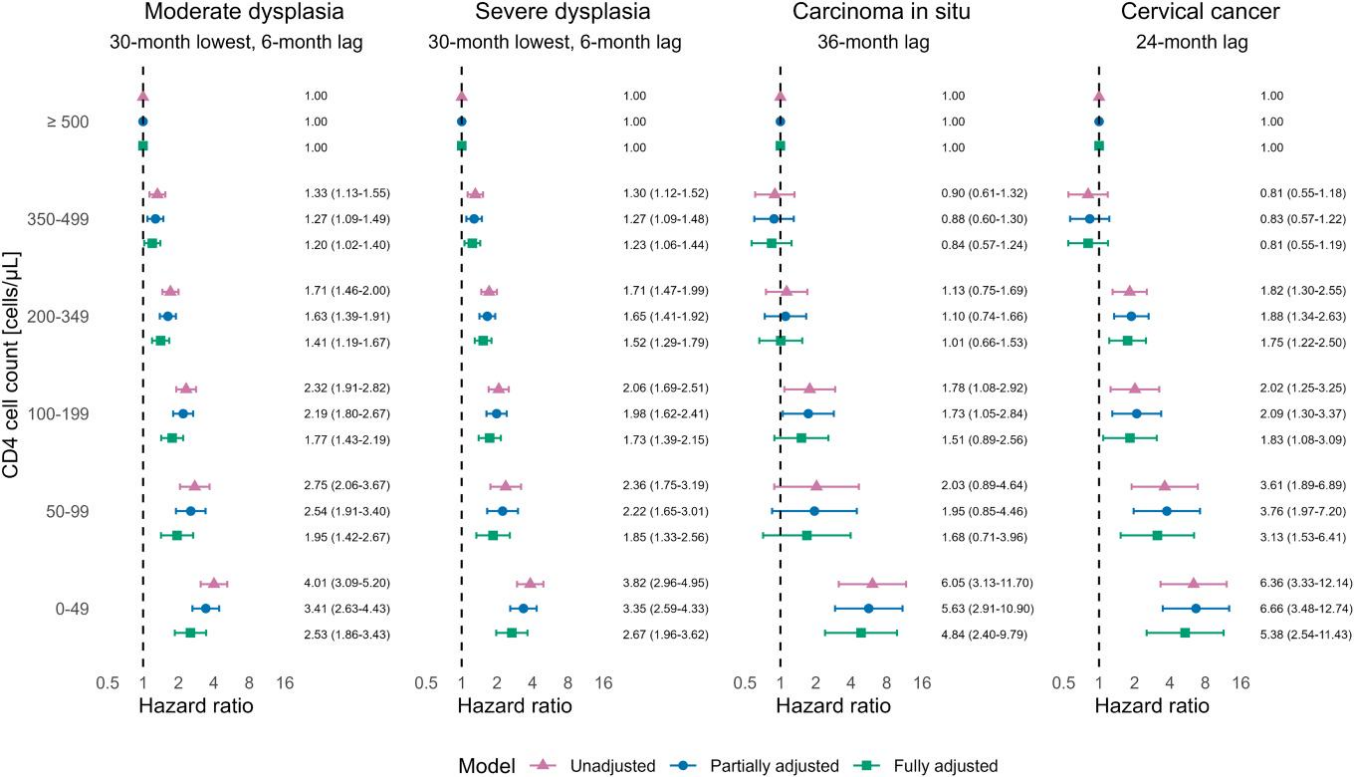
Hazard ratios and 95% confidence intervals for the association of CD4 cell count with the incidence of moderate cervical dysplasia, severe cervical dysplasia, cervical carcinoma in situ, and cervical cancer. CD4 cell count was modeled as a categorical variable, with ≥500 cells/μL as the reference category. The measure used for CD4 cell count differs by the outcome of interest. The partially adjusted model controls for time-updated age, time-updated calendar period, and time-updated history of antiretroviral therapy (no/yes); the fully adjusted model further controls for human immunodeficiency virus ribonucleic acid.

We found that lower CD4 counts were associated with higher rates of cervical precancer and cancer, both before and after adjusting for HIV RNA and the additional factors (Figure 2). There was about a 5-fold increased risk of cervical carcinoma in situ (adjusted HR 4.84, 95% CI 2.40–9.79) and cervical cancer (adjusted HR 5.38, 95% CI 2.54–11.43) among women with a lagged CD4 count < 50 cells/μL compared to those with ≥500 CD4 cells/μL, independently of their HIV RNA. The corresponding associations, using 30-month lowest CD4 count, were somewhat weaker for moderate dysplasia (adjusted HR 2.53, 95% CI 1.86–3.43) and severe dysplasia (adjusted HR 2.67, 95% CI 1.96–3.62).

**Figure 3.**
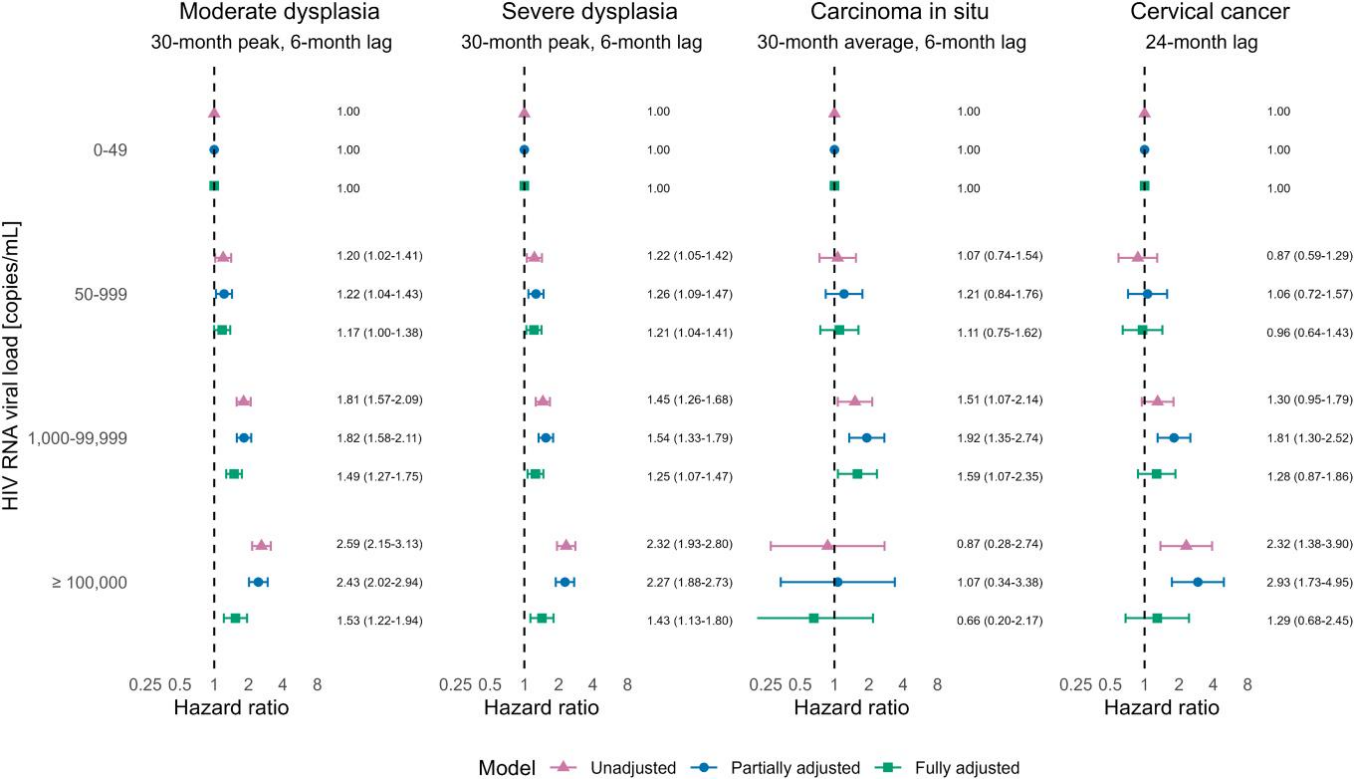
Hazard ratios and 95% confidence intervals for the association of human immunodeficiency virus ribonucleic acid with the incidence of moderate cervical dysplasia, severe cervical dysplasia, cervical carcinoma in situ, and cervical cancer. Human immunodeficiency virus ribonucleic acid was modeled as a categorical variable with 0–49 copies/mL as the reference category. The measure used for human immunodeficiency virus ribonucleic acid differs by outcome of interest. The partially adjusted model controls for time-updated age, time-updated calendar period, and time-updated history of antiretroviral therapy (no/yes); the fully adjusted model further controls for CD4 cell count. Abbreviations: ART, antiretroviral therapy; HIV, human immunodeficiency virus; RNA, ribonucleic acid.

We found a positive association between higher HIV RNA categories and the rates of precancer and cancer in models not accounting for CD4 count; however, this association was attenuated or disappeared after adjusting for CD4 count (Figure 3). For example, the adjusted HR for incident cervical cancer comparing women with an HIV RNA ≥ 100 000 copies/mL versus undetectable viral load decreased from 2.93 (95% CI 1.73–4.95) to 1.29 (95% CI .68–2.45) after controlling for CD4 count. The GVIFs for CD4 count and HIV RNA were <2 for all fully adjusted models, suggesting limited impact from collinearity.

### Sensitivity Analyses

When repeating the model selection process including women with a single ICD-10 diagnosis for cervical cancer, the selected measure for CD4 count remained the same. However, for HIV RNA, it changed from the 24-month lagged time-updated measure to the 30-month average (Supplementary Table 1). The excess risk of cervical cancer in women with lower CD4 counts remained similar when also including women with a single ICD-10 diagnosis for cervical cancer (Supplementary Figure 2).

## DISCUSSION

In this large cohort of women enrolled in a private HIV disease management program in South Africa, we found that both lower CD4 counts and higher HIV RNA were associated with an increased risk of cervical precancer and cancer. Among the CD4 measures examined, 30-month lowest CD4 count best captured the relationship with moderate and severe dysplasia, whereas lagged point-in-time CD4 counts were the most informative for the association with cervical cancer. Human immunodeficiency virus RNA was also associated with precancer and cancer risk, but these associations were attenuated after adjustment for CD4 count, suggesting that HIV-related immunosuppression instead of HIV viremia is the predominant driver of cervical carcinogenesis among WWH. These findings refine our understanding of how HIV-related factors influence cervical disease and can inform cervical cancer prevention efforts. While routine cervical screening remains essential for all WWH and the implementation of risk-based screening approaches would require careful consideration of feasibility and health system constraints, our findings provide clinically relevant insights that may support risk awareness, prioritization of follow-up, and integration of cervical cancer prevention into HIV care.

Key strengths of our study include the large sample size, long follow-up, and comprehensive exploration of multiple representations of immunodeficiency and HIV viremia, with lag periods ranging from 6 to 36 months. This approach allowed us to investigate not only whether HIV-related factors matter but also when they matter most for different stages of cervical carcinogenesis. Another strength is the consistency of our findings across alternative definitions of cervical cancer in sensitivity analyses. Several limitations should be acknowledged. First, our data were derived from a private HIV management program and may not be generalizable to WWH in South Africa's public sector, where quality of care varies [18] and WWH often present later, with more profound immunosuppression, including after the implementation of the Universal Test and Treat policy in 2016 [19]. Less than 16% of South Africa's general population is covered by medical insurance [20], and HIV prevalence is lower among individuals accessing care in the private versus the public sector [21]. Second, outcome ascertainment relied on ICD-10 codes from insurance claims. These claim reports are prone to misclassification and lack the granularity of full histopathology reports [17]. The ICD-10-based classification of cervical precancer differs from the CIN classification system and may not fully align with histopathological diagnoses. Furthermore, using claims-based algorithms to identify high-grade cervical lesions or cervical cancer in Massachusetts, United States, Kim et al obtained positive predictive values of 57% when using a single diagnosis code, improving to 81% when combining multiple diagnoses with procedure codes [16]. We required at least 2 claims for cervical cancer, but our results regarding the association with CD4 counts and HIV RNA were robust in sensitivity analyses that included women with only 1 cervical cancer-related claim. Third, we lacked information on important behavioral and clinical covariates such as ART initiation dates, smoking, sexual history, HPV vaccination, and cervical screening. These factors are known to influence cervical cancer risk and could confound some of the observed associations. Although we tested a wide range of measures for CD4 and HIV RNA, differences in model fit were modest for cervical cancer, indicating that no single measure is definitively superior.

Our results emphasize the role of both past and current immunodeficiency in cervical carcinogenesis. Specifically, the lowest CD4 count within a 30-month window was particularly informative for incident precancer, supporting the concept that severe immunosuppression promotes persistent high-risk HPV infection and lesion development. Although cervical cancer is one of the commonest cancers in sub-Saharan Africa, data from this region are scarce [12]. Our study aligns with a recent cohort study from Botswana, which found that WWH whose nadir CD4 was ≥500 cells/µL had cervical intraepithelial neoplasia grade 2+ (CIN2+) risk comparable to women without HIV, whereas lower nadir CD4 counts were associated with an increased CIN2+ risk [22]. Similarly, a study of previously unscreened WWH in Kisumu, Kenya, found that higher nadir CD4 counts were associated with lower CIN2+ risk [23]. Current CD4 count was protective only among WWH not on ART, possibly because the CD4 count on ART reflects immune recovery after onset of carcinogenesis [23]. In our study, almost all WWH were on ART, precluding analyses stratified by ART status. Our results are also in line with immunological findings: In women with progressive cervical disease, CD4⁺ T-cell responses to HPV antigens are less frequent, indicating that effective CD4-mediated immunity is essential for HPV clearance and lesion control [24]. The attenuated but persistent association between high HIV RNA and cervical precancer after adjustment for CD4 count suggests that uncontrolled HIV replication may play a role in early cervical carcinogenesis beyond its impact on immune depletion. Experimental evidence indicates that HIV-1 proteins such as Tat and gp120 can contribute directly to HPV-mediated carcinogenesis [25, 26]. A recent review notes that these proteins induce epithelial–mesenchymal transition of HPV-infected epithelial cells, thus potentially promoting malignant transformation [27].

Our results highlight the critical importance of sustained immune competence in preventing HPV-related cervical disease among WWH. Early initiation and lifelong adherence to ART remain essential for maintaining high CD4 counts, achieving viral suppression, and reducing cervical cancer risk. Indeed, the studies from sub-Saharan Africa included in Kelly et al's meta-analysis [5] consistently indicate that earlier initiation and effective ART over a prolonged duration can prevent cervical lesion incidence, reduce progression, and promote regression. Our findings also suggest that routine monitoring of CD4 count trajectories may inform cervical cancer risk stratification. Women with a history of profound immunosuppression or evidence of recent immune decline may warrant more intensive cervical screening and follow-up, even if their current CD4 counts are adequate. The study also underscores the value of integrating HIV care and cervical cancer prevention. Women with HIV in South Africa typically engage in regular HIV monitoring, creating opportunities to align CD4 and HIV RNA testing with cervical screening services [28]. Such integration could help identify women at highest risk and ensure they receive timely cervical screening, follow-up, and treatment. In the longer term, expanding HPV vaccination coverage remains essential to reduce the incidence of HPV infection and its sequelae in populations with and without HIV [13, 29, 30]. A 2022 meta-analysis found that even after acquiring HIV, HPV vaccination is safe and elicits a robust immune response [21]. Integrating HPV vaccination into routine HIV care could help improve HPV vaccination uptake among young WWH.

In conclusion, we found that lower CD4 counts and higher HIV RNA were associated with incident cervical precancer and cancer among WWH in South Africa, although the association with HIV RNA was attenuated after adjusting for CD4 count. Lowest CD4 counts in a 30-month window were most informative for the association of immunodeficiency and cervical dysplasia, whereas lagged point-in-time CD4 counts tended to be more informative for invasive cancer. These findings highlight the complex interplay between immune history, current immune function, and viral replication in cervical carcinogenesis. Sustaining high CD4 counts through early ART initiation and effective HIV management, combined with the use of CD4 counts for risk stratification in cervical screening, may help improve cervical cancer prevention among WWH in South Africa.

## Supplementary Material

ciag093_Supplementary_Data

## Supplementary Data

Supplementary materials are available at *Clinical Infectious Diseases* online. Consisting of data provided by the authors to benefit the reader, the posted materials are not copyedited and are the sole responsibility of the authors, so questions or comments should be addressed to the corresponding author.
