# Supplementary material for: Association of Immunodeficiency and HIV Viremia With Cervical Precancer and Cancer Risk Among Women With HIV in South Africa
Source: Clin Infect Dis. 2026 Feb 14;82(5):e976–83. doi: 10.1093/cid/ciag093 (PMC13189670; doi:10.1093/cid/ciag093)

**Supplementary material:**

**Association of Immunodeficiency and HIV Viremia with Cervical Precancer and Cancer Risk among Women with HIV in South Africa**

- **Supplemental Table 1:** Comparison of Akaike’s information criteria (AIC) resulting from Cox regression models using various time-updated measures for CD4 cell count and HIV RNA viral load, with both measures modelled as categorical variables.
- **Supplemental Figure 1:** CD4 cell count (top) and HIV RNA viral load (bottom) trajectories for a random sample of 100 women from the study population. The red lines represent the median trajectory for the study population.
- **Supplemental Figure 2**: Hazard ratios and 95% confidence intervals for the association of CD4 cell count (left panel) and HIV RNA viral load (right panel) with cervical cancer incidence, where only ≥1 C53 ICD-10 code was required for the definition of cervical cancer.

**Supplemental Table 1:** Comparison of Akaike’s information criteria (AIC) resulting from Cox regression models using various time-updated measures for CD4 cell count and HIV RNA viral load. All analyses were performed on a common (by outcome) dataset consisting of person-years-at-risk with available 36-month lagged CD4 cell count and HIV RNA viral load. The measures we chose for the analyses are in bold italics. All models are also adjusted for age, calendar period, and history of ART.

|  | **Outcome** | | | | |
| --- | --- | --- | --- | --- | --- |
|  | **Moderate dysplasia** | **Severe dysplasia** | **Carcinoma in situ** | **Cervical cancer** | **Cervical cancer*** |
| **CD4 cell count measure** |  |  |  |  |  |
| 6-month lag | 24594 | 25423 | 4333 | 4477 | 6533 |
| 12-month lag | 24586 | 25409 | 4343 | 4485 | 6540 |
| 18-month lag | 24582 | 25403 | 4346 | 4474 | 6525 |
| 24-month lag | 24570 | 25399 | 4344 | ***4470*** | ***6521*** |
| 30-month lag | 24583 | 25392 | 4341 | 4486 | 6547 |
| 36-month lag | 24583 | 25410 | ***4332*** | 4496 | 6555 |
| 30-month average, 6-month lag | 24575 | 25409 | 4342 | 4475 | 6528 |
| 30-month percentage time with CD4<200 copies/μL, 6-month lag | 24606 | 25425 | 4342 | 4479 | 6536 |
| 30-month lowest, 6-month lag | ***24555*** | ***25374*** | 4335 | 4480 | 6539 |
| **HIV RNA viral load measure** |  |  |  |  |  |
| 6-month lag | 24634 | 25447 | 4347 | 4492 | 6560 |
| 12-month lag | 24618 | 25440 | 4343 | 4492 | 6558 |
| 18-month lag | 24598 | 25432 | 4342 | 4486 | 6550 |
| 24-month lag | 24584 | 25412 | 4340 | ***4485*** | 6545 |
| 30-month lag | 24594 | 25423 | 4347 | 4487 | ***6543*** |
| 36-month lag | 24599 | 25425 | 4346 | 4491 | 6553 |
| 30-month average on Log10 scale, 6-month lag | 24579 | 25424 | ***4339*** | 4487 | 6547 |
| 30-month percentage time with viral load >50 copies/mL, 6-month lag | 24587 | 25440 | 4342 | 4489 | 6551 |
| 30-month peak, 6-month lag | ***24573*** | ***25409*** | 4341 | 4494 | 6552 |

*sensitivity analysis also including women with a single C53 ICD-10 code for the definition of cervical cancer.

**Supplemental Figure 1:** CD4 cell count (top) and Log10 HIV RNA viral load (bottom) trajectories for a random sample of 100 women from the study population. The red lines represent the median trajectory for the study population**.** HIV RNA viral loads below the detection limit are set to 20 copies/mL, equivalent to 1.3 Log10 copies/mL

**
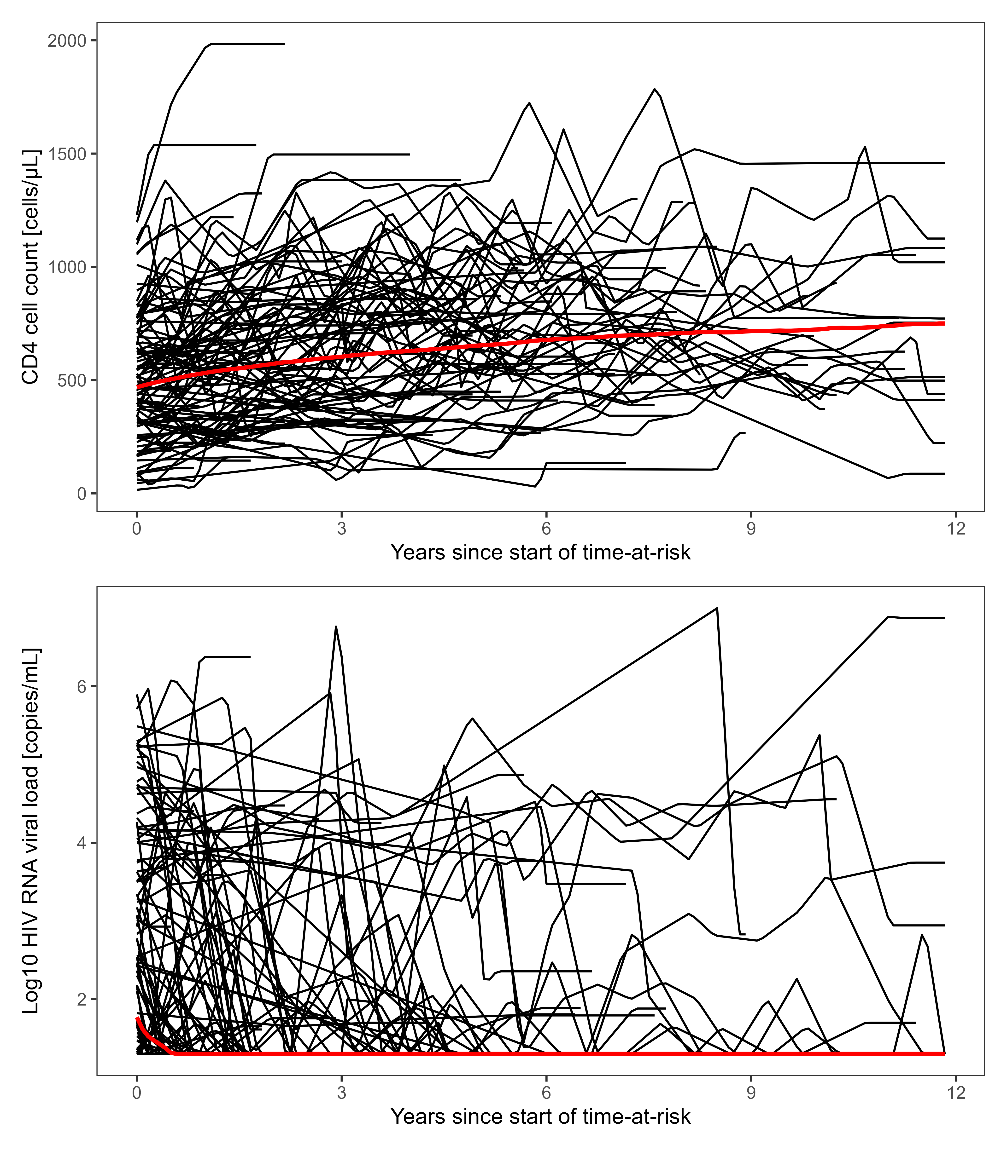
**

**Supplemental Figure 2**: Hazard ratios and 95% confidence intervals for the association of CD4 cell count (left panel) and HIV RNA viral load (right panel) with cervical cancer incidence, where only ≥1 C53 ICD-10 code was required for the definition of cervical cancer. CD4 cell count and HIV RNA viral load were modelled as a categorical variable, with ≥500 cells/μL and <50 copies/mL as reference categories. The partially adjusted model controls for time-updated age, time-updated calendar period, and time-updated history of ART (no/yes); the fully adjusted model further controls for HIV RNA viral load or CD4 cell count.


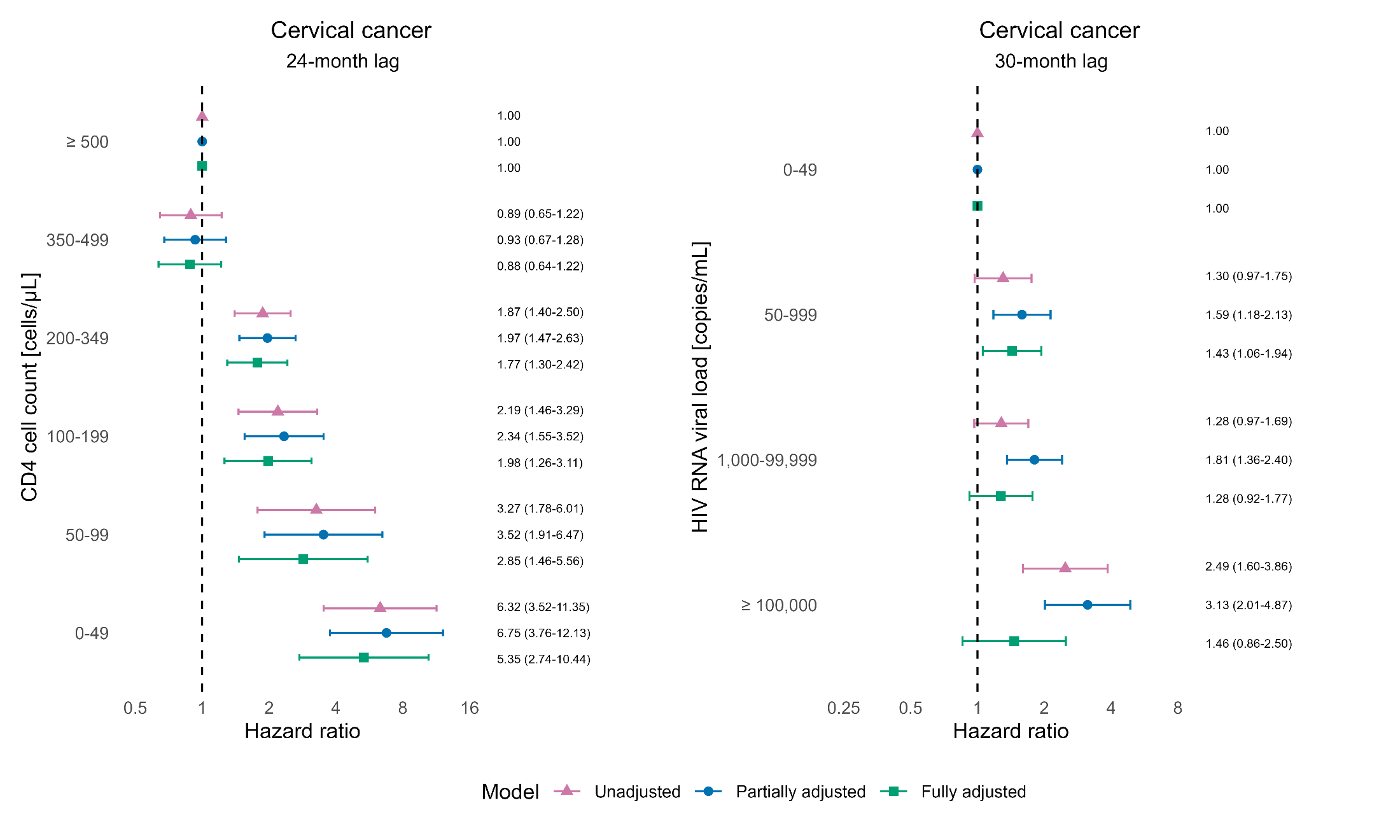

Supplement: ciag093_Supplementary_Data [file ciag093_supplementary_data.docx]
